# Source credibility: a necessary North Star in cancer care

**DOI:** 10.1038/s44276-024-00075-5

**Published:** 2024-08-06

**Authors:** Seamus O’Reilly, Karie Dennehy, Dearbhaile C. Collins

**Affiliations:** 1 CUH/UCC Cancer Centre, Cork University Hospital, Cork, Ireland; 2 Cancer Research@UCC, College of Medicine and Health, University College Cork, Cork, Ireland; 3 Marymount University Hospice and Hospital, Cork, Ireland

Cancer is a lonely illness. For many patients cancer related isolation is relieved by internet based support groups and educational resources. However in the quest to relieve the vulnerability of isolation, the source credibility of what they encounter in internet searches emerges, paradoxically, as an additional vulnerability for them. Consequently as clinicians, the article by Zenone and colleagues [1] featured in the current version of this journal resonated with us, when it was submitted for publication. The authors reviewed the Google listing and Google reviews of 47 prominent alternative cancer clinics in August 2022. They noted that Google rarely declared that these clinics were alternative, and that the clinic approval ratings were high with median scores of 4.5 on a 5 point scale. The clinics were presented as locations to improve or cure cancer in 288 reviews. While reviews also noted concerns about financial exploitation (*n* = 98), poorer outcomes (72), provision of poor care (*n* = 41) and misrepresentation of outcome (*n* = 23) these were counterbalanced by the positive reviews. In our practice, we see patients abandoning potentially curative cancer therapy [e.g., [2]] and forgoing evidence based therapies for these alternative cancer clinics. We have previously documented crowdfunding and other strategies to enable this alternative care to be provided [3]. An additional concern related to these findings are the lack of guard rails for vulnerable patients such as those with intellectual disability or cognitive impairment due to cancer or otherwise. Such groups assume source credibility in the results of their internet searches. Coercion from “loved ones” is a compounding dynamic that we have also encountered.

The results of the present study build on a prior evaluation by the same authors of the advertising patterns of alternative cancer treatments during December 2021 on Meta social media platforms [4]. In this analysis, 25.8% of paid advertisements included direct statements claiming that provider treatment can cure cancer or prolong life. These statements were reinforced by the use of imagery and text content that emulated evidence-based medical providers. The report noted that prior Meta advertisements had disseminated scientifically unsupported public health messages such as anti-vaccine [5] and pro-tobacco content [6]. It noted reports by patients with cancer that they had started to see advertisements for fake cancer cures after their diagnosis [7, 8]. Both studies demonstrate that analogous to the tobacco, firearm and alcoholic beverages industries there is a conflict of interest between such internet platforms and social determinants of public health [9–12].

Both reports have focused on the role of platform providers in the ecosystem of alternative medicine provision. They are published in the backdrop of rising complementary and alternative medicine use globally [13–17] with up to 87% of patients using at least one form of complementary therapy during their cancer treatment. Such use can occur even if it conflicts with medical advice [e.g., [18]] and is present in the context of a lack of engagement by health care professionals on alternative therapy use by their patients [19]. These patients are also vulnerable to the harms of lost financial resources due to in-effective treatments, negative side effects and potential drug interactions from some alternative interventions, encouragement to forego palliative care, and lost and exploited hope. The increasing use of these therapies can be viewed as reflecting the weaknesses of conventional cancer care. The side effects of conventional anti-cancer therapy, and the patriarchal structure of conventional medicine as examples of such weaknesses [20]. In this regard a wider aspect of these reports is the role of medical mistrust in facilitating patient engagement with alternative medicines. Medical mistrust centres on the belief that health care providers, the pharmaceutical industry, academic institutions, or the government as a steward of public health are acting against ones best interests or wellbeing [21].

**Fig. 1:**
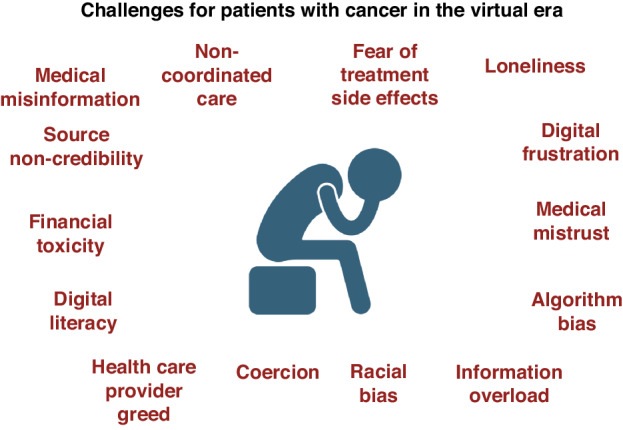
Challenges for Patients with Cancer in the Virtual Era.

The prevalence of medical mistrust was highlighted in vaccine hesitancy rates during the COVID-19 pandemic [22, 23]. It can range from scepticism to belief in conspiracy theories [24]. In cancer care, conspiracy theories are evident both online and off. In 2014 a survey of the American public reported that 37% believed that the Food and Drug Administration intentional suppressed natural cures for cancer at the behest of the pharmaceutical industry [25, 26]. “Big Pharma” is a constant refrain underlying much cancer information [27]. Such refrains are reinforced by the egregious behaviour of companies such as Purdue Pharma and it is associated, medically qualified Sackler family members [28, 29]. The entanglement of the medical community with the pharmaceutical industry compounds this perception [30]. The consequence of this ecosystem of conspiracy theories is significant for patients, including ones we have encountered in our clinical practice (Fig. 1).

Patients who subscribe to alternative therapies are twice as likely to die in the same period as those who rely on conventional therapies either due to delays in treatment or rejection of conventional therapy [31].

What are the implications of Zenone and colleagues paper to our clinical practice today? In the first instance, we need to be cognisant of the growing scale of misinformation that is present for patients and their families. Articles such as this paper can support awareness and dissemination. Moreover, increased professional engagement and discussion is required. Studies cited in this editorial demonstrate the significant prevalence of complementary and alternative therapy use in our community. A call to action is needed to scrutinise the current growth and the systems used to promote them which includes social media, podcasts in addition to organisational websites.

The issues raised by Zenone and colleagues [1, 4] and others [25] require statutory intervention to protect patients and their families, and ensure source credibility in their internet interactions. This should include consequences for service providers. We are cognisant of the fact that search engine providers can’t exclude all misinformation but integrating better signposting to reputable sources of information could be a first step in addressing this issue. Solutions could include a global agreement of information verification indicators (analogous to the X/twitter blue tick), artificial intelligence recognition of the need for caution with website claims, and expert validated Google reviews.

In our professional lifetimes we have witnessed the positive impact of the internet on cancer care. For millions of healthcare providers it is their “go to” source for treatment planning for patients. For patients and their families it is an essential repository for access to information, access to charitable support services, access to clinical research options and access to patient communities who have invaluable, shared, lived experiences. Ensuring that the internet remains a trusted resource for ALL is in everyone’s interest, including those who profit from it.
